# Optical Modification of a Nanoporous Alumina Structure Associated with Surface Coverage by the Ionic Liquid AliquatCl

**DOI:** 10.3390/mi15060739

**Published:** 2024-05-31

**Authors:** María Cruz López-Escalante, Mª Valle Martínez de Yuso, Ana L. Cuevas, Juana Benavente

**Affiliations:** 1 The Nanotech Unit, Laboratorio de Materiales y Superficies, Departamento de Ingeniería Química, Facultad de Ciencias, Universidad de Málaga, 29071 Málaga, Spain; mclopez@uma.es; 2 Laboratorio de Espectroscopía de Rayos X, Servicios Centrales de Apoyo a la Investigación (SCAI), Universidad de Málaga, 29071 Málaga, Spain; mvyuso@uma.es; 3 Unidad de Nanotecnología, Servicios Centrales de Apoyo a la Investigación (SCAI), Universidad de Málaga, 29071 Málaga, Spain; analaura.cuevas@uma.es; 4 Departamento de Física Aplicada I, Facultad de Ciencias, Universidad de Málaga, 29071 Málaga, Spain

**Keywords:** nanoporous alumina, ionic liquid, photovoltaic

## Abstract

This manuscript analyses changes in the optical parameters of a commercial alumina nanoporous structure (Anodisc^TM^ or AND support) due to surface coverage by the ionic liquid (IL) AliquatCl (AlqCl). XPS measurements were performed for chemical characterization of the composite AND/AlqCl and the AND support, but XPS resolved angle analysis (from 15° to 75°) was carried out for the homogeneity estimation of the top surface of the ANDAlqCl sample. Optical characterization of both the composite AND/AlqCl and the AND support was performed by three non-destructive and non-invasive techniques: ellipsometry spectroscopy (SE), light transmittance/reflection, and photoluminescence. SE measurements (wavelength ranging from 250 nm to 1250 nm) allow for the determination of the refraction index of the AND/AlqCl sample, which hardly differs from that corresponding to the IL, confirming the XPS results. The presence of the IL significantly increases the light transmission of the alumina support in the visible region and reduces reflection, affecting also the maximum position of this latter curve, as well as the photoluminescence spectra. Due to these results, illuminated I–V curves for both the composite AND/AlqCl film and the AND support were also measured to estimate its possible application as a solar cell. The optical behaviour exhibited by the AND/AlqCl thin film in the visible region could be of interest for different applications.

## 1. Introduction

The fabrication of nanoporous alumina structures (NPASs) by the electrochemical anodization of aluminium foils via a two-step anodization process [1] allows for thin films with a highly homogeneous ordered cylindrical nanoporous structure to be obtained, practically without tortuosity, that is, with nano-channels perpendicular to sample surfaces, which are of great interest for different applications. Initially, these nanoporous structures were used as templates for the fabrication of nanotubes, nanodots, or nanowires [2,3], but due to their high thermal and chemical stability, as well as their non-toxic and biocompatibility properties, they were also considered (after removing the bottom aluminium surface) for environmental, biotechnological, or biomedical applications (protein filtration, water desalination, diffusion controlling systems, virus detection, biomolecules recognition, or separation processes [4,5,6,7,8,9]). Moreover, the electropositive character of the NPASs also provides them with a certain ion selection character (anion exchanger) in the case of samples with a low pore size and porosity [10,11,12]. On the other hand, NPASs with modulated diameters or branched channels, that is, with an asymmetric structure, can also be obtained [13,14]. This point could be of interest since nanopore length seems to affect the sensing applications of NPASs, but also when used as nanofilters or membranes (higher flow for similar retention) [15,16]. On the other hand, it is worth noting the recent use of alumina nanohole arrays for the adsorption/desorption of loaded lipid nanoparticles to improve storage at the nanoscale for therapeutics disciplines [17].

Typical electrolytes used for NPASs fabrication are sulphuric, oxalic, and phosphoric acids, obtaining samples with average pore sizes/interpore distances around 30 nm/65 nm in the case of sulphuric acid, 50 nm/105 nm for oxalic acid, and 300 nm/490 nm for phosphoric acid [18], although the use of other electrolytes, such as malonic acid or even electrolyte mixtures, is also reported [19]. Moreover, the easy pore size and interpore distance modification (consequently, pore density or porosity) of the NPASs depending on the anodization conditions (electrolyte solution, but also anodization voltage, time, or temperature), along with the simplicity and relatively low fabrication cost, are two factors that have favoured their used in many nanotechnology applications. Particularly, the specific optical properties of the NPASs such as luminescence (photo and electrical luminescence) and photonic crystal characteristics, associated mainly with pore size and porosity (that is, with fabrication conditions), are of great interest in different technological areas, and various reviews describing NPASs fabrication and optical characterizations have lately been published [20,21]. In fact, it was reported that NPASs fabricated with oxalic acid present a rather well-defined photoluminescence spectra with a maximum of around 450 nm (430–460 nm depending on pore size), while wider curves are obtained for films obtained with malonic or phosphoric acids [20,22]. 

On the other hand, the possibility of the easy functionalization/modification of NPAS surfaces by different techniques is another significant point of interest considering NPAS applications. In particular, different types of changes (optical, electrochemical, and interfacial) in NAPSs associated with diverse surface modifications, such as the coating of ceramic layers by atomic layer deposition (ALD) or sputtering scattering, surface coverage by macromolecules or different room-temperature ionic liquids (ILs in shorted form), among others, have already been reported [23,24,25,26,27,28,29,30]. In fact, optical changes due to the surface modification of alumina nanoparticles or asymmetric NPASs with different imidazolium-based ILs (the imidazole ring consisting of five carbon atoms and two nitrogen atoms) were described and applied to energy conversion devices [31,32]. In this context, changes in the physicochemical properties of ILs in contact with charged surfaces, depending on the type of IL ions or the solid surface chemistry, have also been reported [32].

ILs are compounds with very low vapour pressure, remaining in liquid state for temperatures below 100 °C, which are typically comprised of an organic cation paired with an inorganic/organic anion [33]. ILs are of great interest nowadays due to their significant properties, such as high thermal and chemical stability, low volatility, and good electrical conductivity, but they are also non-flammable compounds and, due to their good ability to dissolve a wide range of compounds, they are considered green solvents. Moreover, since an adequate election of the cation/anion pair allows for the selection of the most appropriated combination for a specific application, ILs are considered to be novel and promising materials in many areas, such as separation processes, optical sensors, or biomedical applications [34,35,36,37,38]. In particular, ILs such as trioctylmethylammonium chloride (or AliquatCl) and its derivatives (trioctylmethylammonium nitrate or trioctylmethylammonium thiocianate) are being used in the fabrication of polymer inclusion membranes (PIMs) for the removal of pollutants from natural waters (small organic molecules or metallic species), acting as extractants or carriers, but also improving the mechanical stability of PIMs [39,40,41,42,43,44]; other ILs have been incorporated into the structure of Nafion membranes for the reduction in gas and methanol crossover or to improve the membrane stability at a temperature higher than 80 °C [45,46,47,48,49]. Due to the particular characteristics of ILs when compared to conventional electrolytes, they are also used for electrochemical devices (batteries, capacitors, or fuel cells), electrodeposition, catalysis, renewable energy generation, and storage processes [50,51,52,53,54,55]. Moreover, ILs have lately begun to be considered as novel electrolytes in different types of solar cells (dye-sensitized solar cells, polymer solar cells, or thin film solar cells) [56,57,58,59]. 

This work analyses optical changes in a commercial asymmetric nanoporous alumina support (AND) as a result of surface coverage with the IL AliquatCl (a quaternary ammonium salt containing a mixture of C_8_ and C_10_ chains [60]). Chemical surface analysis of both the composite AND/AlqCl film and the AND support was performed by X-ray electron spectroscopy (XPS) to verify surface coverage, but angle-resolved XPS analysis (after sample use) was also carried out for top-surface layer homogeneity estimation. Different non-invasive contactless optical techniques such as spectroscopic ellipsometry, light transmission/reflection, or photoluminescence have been used for characterization of the samples. Spectroscopic ellipsometry, an optical technique, has allowed for the evaluation of the characteristic optical parameters (refraction index, extinction coefficient, or dielectric constant) of the composite AND/AlqCl thin film and the AND support. Light transmission and reflection measurements (for wavelength ranging between 200 nm and 2000 nm) provided information for ultraviolet (UV), visible (V), and near-infrared (NIR) regions, showing significant changes caused by the AlqCl IL coverage, but also when the support was covered by other liquids with different characteristics. Modification of the photoluminescence spectra of the nanoporous alumina support due to the surface coverage by the AlqCl IL was also established. Moreover, taking into account the transmission/reflection results, the possible application of the composite AND/AlqCl thin film to solar cell devices was also considered, and the I-V curves for both the AND/AlqCl and the AND samples were measured. The efficiency increase showed for the composite AND/AlqCl thin film when compared with the alumina support (~30%) confirms its possible application in solar cell technologies.

## 2. Materials and Methods

### 2.1. Materials

A commercial asymmetric nanoporous alumina structure (or membrane) obtained by the electrochemical anodization method from Whatman (Maidstone, UK) (Anodisc^TM^ or AND), with a surface of 0.95 cm^2^, was used as a support. The AND geometrical characteristics (according to supplier) are as follows: 60 μm thickness, 10 nm nominal pore radius, and 25–30% porosity for the denser surface, but 100 nm pore radius and 40% porosity for the opposite surface [11]. Moreover, the AND support seems to present a weak cationic character [10,11]. SEM micrographs of the AND surfaces are given as Supplementary Information (Figure S1).

The room-temperature ionic liquid trioctylmethylammonium chloride (AliquatCl or AlqCl in shorter form, but commercially Aliquat 336; chemical formulae, C_25_H_54_ClN)), a quaternary ammonium salt consisting of a mixture of C_8_ (octyl) and C_10_ (decyl) chains (C_8_ predominating) from Sigma-Aldrich (Saint Louis, MO, USA) was used for surface coverage of the denser surface of the AND support by pouring a certain amount of the IL able to cover the total support area. This sample will be hereafter named AND/AlqCl. The basic physicochemical characteristics of AlqCl are as follows: molecular weight, 404.2 g/mol; density, 0.88 × 10^3^ kg/m^3^; viscosity, 1500 mPa.s (at 30 °C); and refractive index, 1.447 [61,62].

It should be pointed out that the two components of the sample studied (the support and the IL) are commercials, simplifying the modification procedure and reducing preparation time. On the other hand, according to previous results performed with the same AND support but different ILs (three different imidazolium-based ILs) by means of density functional theory (DFT) [30], alumina-IL interactions correspond to the formation of non-covalent bonds, whose origin is purely coulombic.

### 2.2. Chemical Surface Characterization by XPS

A Physical Electronics Spectrometer (PHI 5700) with X-ray MgK_α_ radiation (15 kV, 300 W and 1253.6 eV) as the excitation source was used for chemical surface characterization of both the AND/AlqCl composite thin film and the AND support. XPS measurement using conventional equipment was performed for the AND/AlqCl composite thin film due to the high viscosity of the ionic liquid [61]. For comparison reasons, XPS measurements of the opposite surface of each sample, named as AND/AlqCl(op) and AND(op), were also performed.

For these measurements, a concentric hemispherical analyser, operating in the constant pass energy mode at 29.35 eV, was used for recording the high-resolution spectra at a take-off angle of 45° (equipment optimum). The diameter of the analysed area was 720 µm, and each spectral region was scanned several times in order to use a good signal (low noise contribution), and the binding energies were determined with respect to the position of the adventitious C 1s peak at 285.0 eV. The residual pressure in the analysis chamber was maintained below 5 × 10^−^^7^ Pa during data acquisition. Binding energies (accurate ±0.1 eV) were determined with respect to the position of the adventitious C 1s peak at 285.0 eV. Shirley-type background and Gauss–Lorentz curves were used to determine the binding energies (B.E.) following the methodology described in detail elsewhere [63].

After the optical characterization measurements, new XPS analysis for the AND/AlqCl composite thin film at five different take-off angles (ϕ = 15°, 30°, 45°, 60°, and 75°), angle-resolved XPS analysis, and ARXPS were also performed to detect possible top surface contamination (~10 nm thickness [64]) as a result of sample manipulation. 

### 2.3. Optical Characterization

Spectroscopic ellipsometry (SE) measures two characteristic parameters (angles Ψ and Δ), which are related with differential changes in amplitude and phase between incident and reflected light waves [65], and a scheme for both a monolayer and a two-layer system is given as Supplementary Information (Figure S2). SE measurements were carried out with a spectroscopic ellipsometer (Sopra-Semilab GES-5E, Sopra-SemiLab GES-5E, Paris, France) for wavelengths ranging from 250 nm to 1250 nm. Since ellipsometric quantities can be influences by surface roughness or contamination [66], measurements were performed at two incident angles (ϕ_i_ = 65° and 75°) to detect such effects on the SE results. WinElli software v.2.2 from Sopra-Semilab (Paris, France) was used for data analysis and fittings. From Ψ and Δ values, a Cauchy dispersion model relation was used for determination of the optical parameters [67].

Transmittance and reflectance curves were recorded with a Varian Cary 5000 spectrophotometer (Agilent Technologies, San Francisco, CA, USA) provided with an integrating sphere of Spectralon for a wavelength in the range from 250 nm to 2000 nm, allowing for optical information on visible (V) but also near-infrared (NIR) and UV regions to be obtained. Measurements were performed with the incident light normal to sample surface.

The photoluminescence (PL) spectrum was measured at room temperature using a photoluminiscence microscope from HORIBA Scientific (LabRam PL Microscope, Paris-Saclay, France) with a laser excitation light λ_ex_ of 475 nm and 0.028 mW power. 

### 2.4. I-V Curves Measurements

An ABA class LED solar simulator (LSH-7320, Newport Company, Irvine, CA, USA) from Oriel S.A. was used for measuring I-V under standard conditions according to IEC 60904-9 (1000 W/m^2^ irradiance at 25 °C and incident spectrum AM1.5G) [68]. I-V curves for both the composite AND/AlqCl sample and the AND support were measured with a commercial monocrystalline silicon solar cell (total area 243.36 cm^2^, but the radiation of the solar simulator only reaches a circular area of 12.566 cm^2^). The I-V curve for each sample was measured four times, under similar conditions, to reduce measurement uncertainty, and average values of the estimated parameters will be provided.

## 3. Results

**Figure 1:**
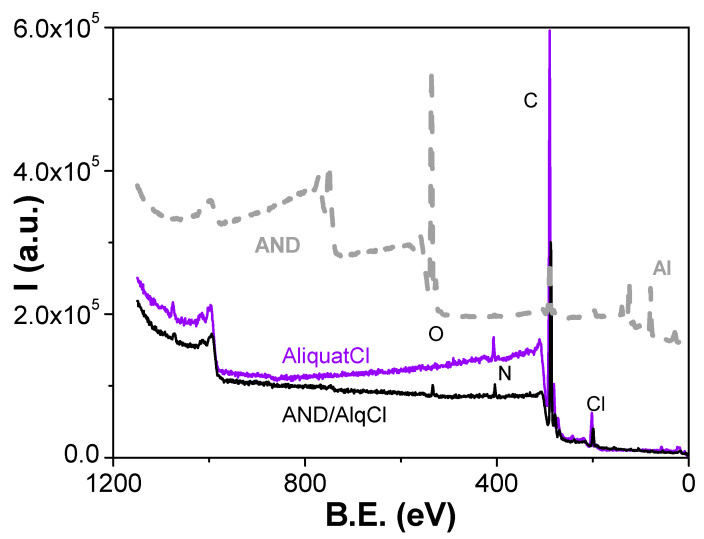
Survey spectra for the composite AND/AlqCl film, the AND support, and the IL AlqCl.

Information on surface chemical changes due to the AlqCl coverage of the nanoporous alumina support was considered previously to the optical characterization of the composite AND/AlqCl thin film. Figure 1 shows the survey XPS spectrum obtained for the AND/AlqCl sample but, for comparison reasons, the spectra for the AND support and the IL AlqCl are also indicated [44]. These spectra were obtained at the equipment optimum take-off angle (45°). The presence of carbon, nitrogen, and chlorine, the characteristic elements of the IL AlqCl, can clearly be observed on the surface of the AND/AlqCl sample, which is an indication of its surface coverage by the IL; moreover, the presence of oxygen, an element associated with the AND support, is also clearly detected in the spectrum of the AND/AlqCl sample.

**Figure 2:**
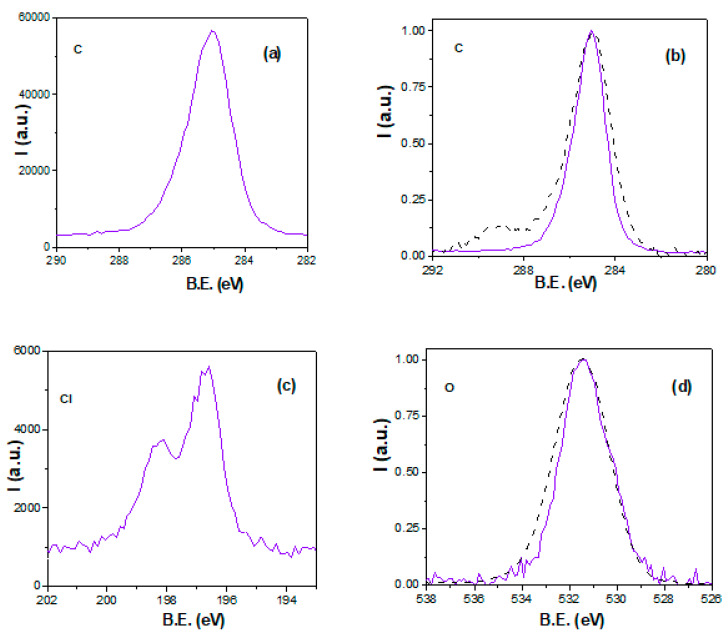
Core-level signals: (**a**) carbon for AND/AlqCl film (solid violet line); (**b**) normalized carbon for the AND/AlqCl film (violet solid line) and the AND support (black dashed line); (**c**) chlorine for the AND/AlqCl film (solid violet line); (**d**) normalized oxygen for the composite AND/AlqCl film (violet solid line) and the AND support (black dashed line).

Figure 2 shows the core-level signals of different elements detected on the surface of the films. The carbon spectrum (Figure 2a) shows a very-well-defined, rather narrow, and symmetric peak at the binding energy (B.E.) of 285.0 eV for the composite AND/AlqCl film, associated with the aliphatic carbon chain of the AlqCl IL (C-C bond), although a weak shoulder at the B.E. of 286.0–286.3 eV (C-N bond) can also be observed. This core-level signal is very similar to that previously obtained for the IL AlqCl, differing clearly from that determined for the alumina AND support, as it can be observed in Figure 2b, where a comparison of the normalized spectra obtained for both samples is presented. Although carbon is not an alumina element, it is a pollution element currently detected in NPASs due basically to the electrochemical anodization process and bottom layer opening [18]. For that reason, the carbon spectrum of the AND shows itself to be slightly wider with C=C, C-O-C, and O-C=O bonds (typically attributed to contamination). However, the chlorine spectra (Figure 2c) for the AND/AlqCl show the typical signal for ionic chlorides [69], as was already obtained for the IL AlqCl [44]. The normalized oxygen spectra for the AND/AlqCl and the AND samples show a practically symmetric peak (B.E. ~531.5 eV) associated with the Al_2_O_3_, but the contamination contribution (C-O-C bond) is also detected in the signal for the AND support. 

The area of the curves shown in Figure 2 allows for the determination of the atomic concentration percentage (A.C. %) of the corresponding elements, and the values obtained for the composite AND/AlqCl film and the AND support are indicated in Table 1. These results indicate an almost complete surface coverage of the support by the AliqCl IL; for comparison reasons, the A.C. % for the IL AlqCl (or C_25_H_54_ClN) is also shown in Table 1. Moreover, the A.C. % for the elements detected on the opposite surfaces of both samples (AND/AlqCl(op) and AND(op)), as well as the ratios between the A.C. (%) of some elements, are also shown in Table 1. Other elements associated with contamination are also indicated as the table food note. In particular, a small percentage of silicon, attributed to contamination from the glass bottle where the IL AlqCl was maintained (SiO_2_), was also detected on the surface of the AND/AlqCl film, while the presence of phosphorous on the surface of the nanoporous alumina support is associated with contamination during the anodization fabrication process [25]. The values indicated in Table 1 seem to indicate a slight excess of carbon and nitrogen, two elements commonly associated with environmental contamination as well as surface oxidation, but also the percolation of AlqCl through the nanopores of the alumina support.

**Figure 3:**
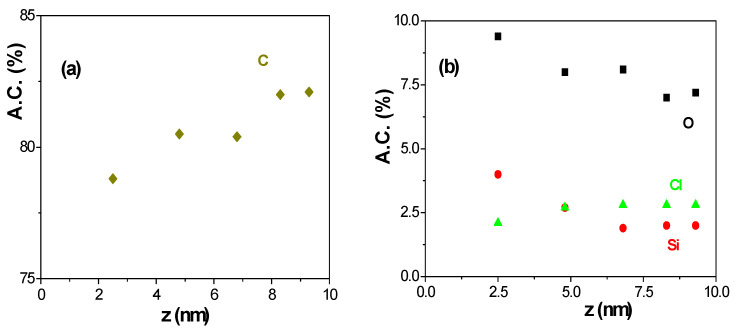
Depth evolution of A.C. % of different elements on the surface of the composite AND/AlqCl film determined by ARXPS analysis: (**a**) carbon (♦), (**b**) oxygen (■), silicon (●), chlorine (▲).

As it was already indicated, the XPS results for the composite AND/AlqCl thin film previously analysed correspond to data values determined at the equipment optimum take-off angle (45°). However, gradual information on the top film surface (<10 nm) can be obtained by in-depth analysis varying the take-off angle (ϕ) considering the relation between the escape length and the photoelectrons mean free path (z and λ_pe_, respectively): z ≤ 3λ_pe_ sin(ϕ) [64]. Figure 3 shows the in-depth variation in the atomic concentration percentages of some elements detected in the top part of the AlqCl IL coating layer (carbon, chlorine, oxygen, and silicon; two are characteristics of the sample, and the other two are associated with contamination). These results show an increase in the carbon concentration percentage by going deeper into the IL layer (Figure 3a), which could be related to top surface pollution reduction; this point is supported by the results shown in Figure 3b, where a reduction in silicon and oxygen percentages (contamination elements already associated with SiO_2_) is observed, while chlorine A.C. % remains practically constant. These results seem to indicate the rather good uniformity of the thin top layer of the IL, with a practically constant value of all the chemical elements for thickness > 7 nm. It should also be indicated that practically no differences were found between these results for ϕ = 45° and those indicated in Table 1 (also performed at 45° previously to optical characterization).

**Figure 4:**
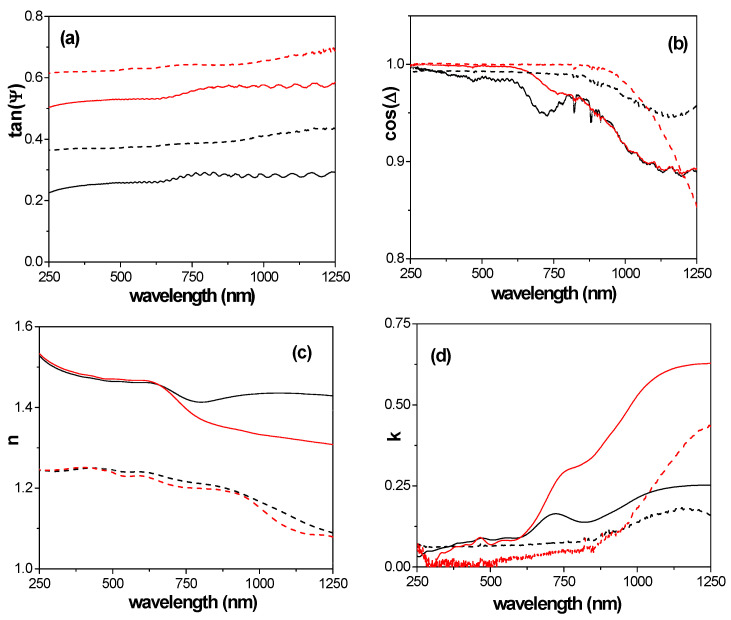
Wavelength dependence of (**a**) tan(Ψ); (**b**) cos(∆); (**c**) refraction index; (**d**) extinction coefficient at two light incident angles (ϕ) for the composite AND/AlqCl film (solid lines) and the AND support (dashed lines). ϕ = 65° (black lines), ϕ = 75° (red lines).

Spectroscopic ellipsometry (SE) is a technique used for the optical characterization of thin films since it provides information on optical parameters (refraction index, extinction coefficient, or dielectric constant) or layer thickness [63,67]. Typical experimental SE values are angles Ψ and Δ, which are related with differential changes in light amplitude and phase by the following expression [65,67]:

tan(Ψ)e^iΔ^ = r_p_/r_s_ (1)

where r_p_, y, and r_s_ are the amount of light reflected in the perpendicular and parallel planes of incidence. The variation in the SE experimental data, tan (Ψ) and cos (Δ), with wavelength (λ) for the composite AND/AlqCl film and the AND support, at the two light incident angles measured, is shown in Figure 4a,b. Differences depending on both the incident angle and sample can be observed, making the effect of surface material more significant for tan(Ψ). From tan(Ψ) and cos(Δ) values, the refraction index (n) and extinction coefficient (k) were determined using equipment software, and their dependence with wavelength is shown in Figure 4c,d.

The values in Figure 4c show an increase of around 18% in the refraction index of the composite AND/AlqCl film in the UV–visible region when compared with the AND support. Practically no differences depending on the light incident angle were observed in such a region for both the refraction index and the extinction coefficient for the composite AND/AlqCl sample, but it significantly affects the values in the NIR (reduction of ~10% for n values and almost three times increase for k one), as it can be observed in Figure 4c,d. The results in Figure 4c allow us to determine the following average value, for the whole wavelength range, and for the refractive index of the AND/AlqCl composite film, <n> = 1.44 ± 0.02, which correspond practically to that for the AlqCl IL and, consequently, is a confirmation of support coverage by the IL. Moreover, since most of the tabulated values of the refraction index for ILs are determined at a constant wavelength of 589.3 nm (92% [70]), this result confirms its validity for a wider range of wavelength. The average value for the AND support, <n> = 1.18 ± 0.11, is clearly lower than the theoretical value for solid alumina (1.76 [62]), but also for that determined for other symmetric nanoporous alumina films with different pore sizes: <n> = 1.54 ± 0.06 (for a sample with pore size around 10 nm) or <n> = 1.52 ± 0.02 (for a sample with ~80 nm pore radius), but with similar porosity (~10%) and thickness (~60 μm) [71]. Consequently, the high porosity of the AND support seems to be responsible of both the low value of the refraction index and the absence of oscillations, that is, the absence of photonic crystal characteristics exhibited by other NPASs with pore side ranging between 20 nm and 60 nm [25,71].

**Figure 5:**
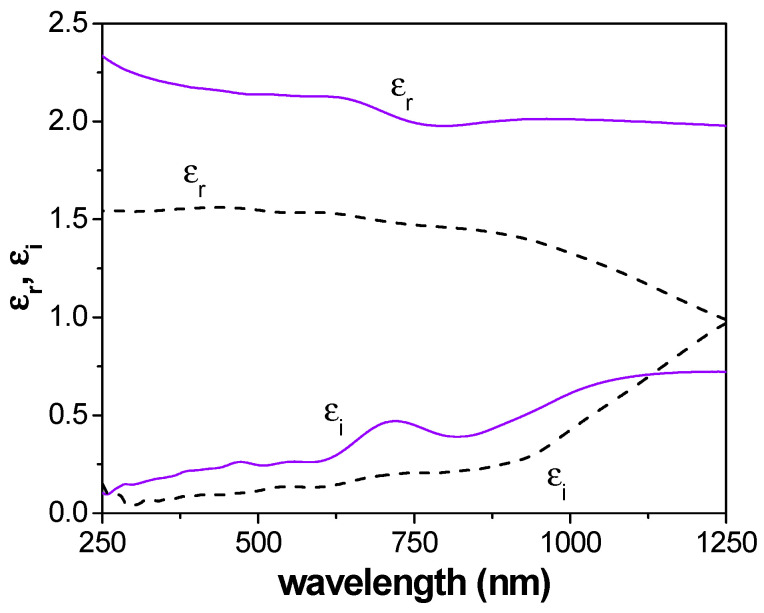
Wavelength dependence of the real (ε_r_) and imaginary (ε_i_) parts of the dielectric constant. AND/AlqCl film (solid violet lines); AND support (dashed black lines).

The SE results also provide information on the real and imaginary parts (ε_r_ and ε_i_, respectively) of the dielectric constant, taking into account the following: ε = (ε_r_ + i ε_i_) = (n + i·k)^2^ [65]. Figure 5 shows the wavelength dependence for ε_r_ and ε_i_ for both AND/AlqCl and AND films for an incident angle of 65°. The values in Figure 5 show an increase of around 38% for ε_r_ and 80% for ε_i_ associated with the AlqCl IL coverage of the AND support. Since the real part of the dielectric constant is related to material polarization and the imaginary part is associated with the dissipation of energy, these results could be of interest for energy conversion applications.

**Figure 6:**
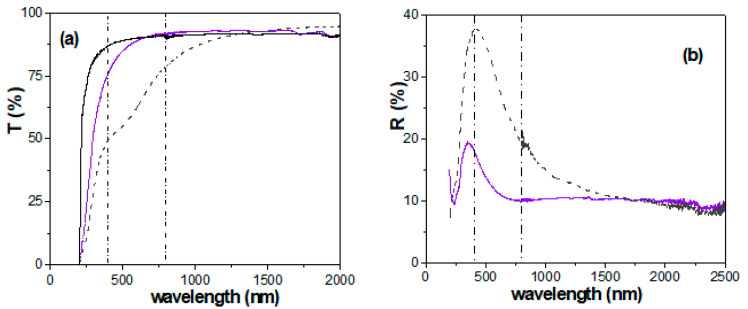
(**a**) Light transmission percentage as a function of the wavelength: composite AND/AlqCl film (solid violet line) and AND alumina support (dashed black line); for comparison, T (%) values for the Al-Sf nanoporous alumina structure (11 nm pore size and 10% porosity [70]) are also shown (solid black line). (**b**) Light reflection percentage as a function of the wavelength for composite AND/AlqCl film (solid violet line) and AND alumina support (dashed black line).

Light transmittance and reflection also give optical information of interest on materials and modifications. Figure 6a shows the variation in the light transmission percentage, T (%), with wavelength for AND/AlqCl and AND thin films. Significant differences in T (%) values in the visible region (delimited by the two dashed–dotted vertical lines) can be observed, making the values for the composite AND/AlqCl film significantly higher than those for the AND support. This fact seems to be related with the relatively high porosity of the alumina support, according to the results obtained for an experimental nanoporous alumina symmetric structure with a similar total thickness (Al-Sf sample, average pore size of 11 ± 2 nm [71], similar to that of the denser AND surface, where the IL was deposited) but lower porosity (~10%); however, neither the lower porosity nor the coating with the IL seem to affect the band gap of the samples. As can be observed, the composite AND/AlqCl film presents a very high constant transmission value for wavelengths ranging between 800 nm and 2000 nm (~92.5%), being slightly higher in the figure (94.3%) for the AND support only in the highest interval of frequency (1500 < λ (nm) < 2000). It should be indicated that similar light transmission vs. wavelength dependence was obtained when the AND support was covered with other liquids with very different characteristics (ILs OMIMPF_6_ or distilled water), as it is indicated in the Supplementary Information (Figure S3a). As it can be observed, very similar values are obtained in the range of frequency between 300 nm and 2000 nm independently of the IL, but a certain difference seems to exist at lowest frequencies. According to these results, it is possible to select the percentage of light transmission for a composite liquid/alumina structure depending on the chosen liquid. It should be indicated that slightly higher T (%) values for the IL AlqCl were also obtained when compared with other imidazolium-based ILs (such as BMIMPF_6_) independently of the support (nanoporous alumina or regenerated cellulose [72]).

The effect of AlqCl coverage on the light reflection percentage can be observed in Figure 6b, which shows a comparison of the values obtained for both AND/AlqCl and AND thin films. These results show a clear reduction in the light reflection percentage of the alumina support for wavelengths ranging between 250 nm and 1500 nm, as a result of surface coverage with the IL AlqCl. This point might be of interest in solar anti-reflection devices. Moreover, AlqCl IL coverage also causes a slight shift to lower wavelength values (from 405 nm to 385 nm) in the maximum of the reflection curve. Differences in light reflection, depending on the liquid chosen for the AND support surface coverage, were also obtained, and they can be observed in the Supplementary Information (Figure S3b). 

**Figure 7:**
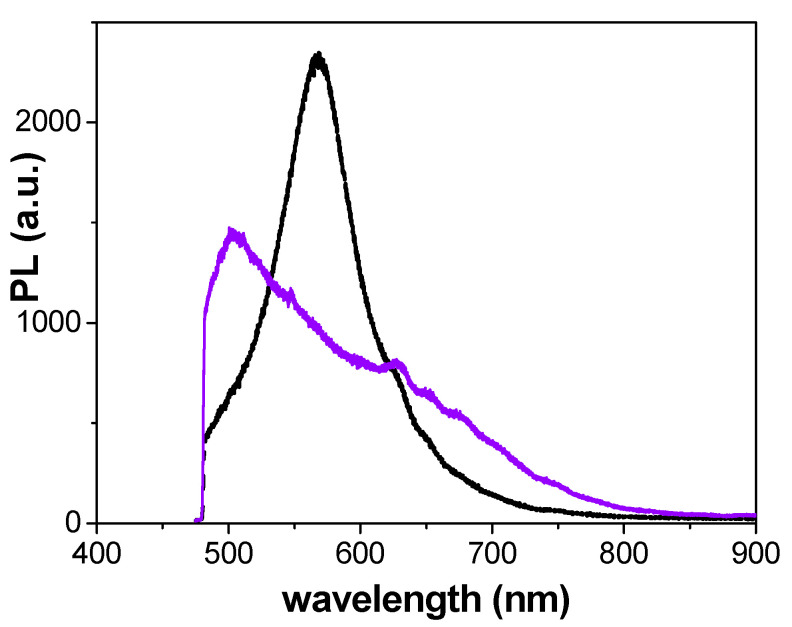
PL spectra for the composite AND/AlCl film (solid violet line) and the AND support (solid black line).

As it was already indicated, photoluminescence (PL) has become a significant property of thin nanoporous alumina structures, since it allows for their use in different applications of technological interest. The intensity of the PL spectra depends on different factors (time of electrolyte and composition, anodization time, temperature, etc.), which usually also affects the pore size and porosity [14,18,19]; in many cases, the increase in these two latter geometrical parameters seems to decrease the PL response of NPASs [19]. Moreover, differences in the PL spectra for similar alumina supports depending on surface materials (different ceramic oxides deposited by atomic layer deposition technique or surface coverage by imidazolium based ILs), which affect both intensity and/or maximum position, have already been reported [14,30,66]. Consequently, the photoluminescence measurement for the composite AND/AlqCl film was also performed and compared with that obtained for the AND support, and these results are shown in Figure 7 (λ_ext_ = 450 nm). As expected, the surface coverage of the nanoporous alumina support by the AlqCl IL reduces the area of the PL curve (~6%), but it also affects the maximum position (from 578 nm to 505 nm). This latter change could be of significant interest, since it shows an increase in the part of the PL curve associated with more energetic photons in the electromagnetic spectrum, which is a key factor for solar cell applications.

**Figure 8:**
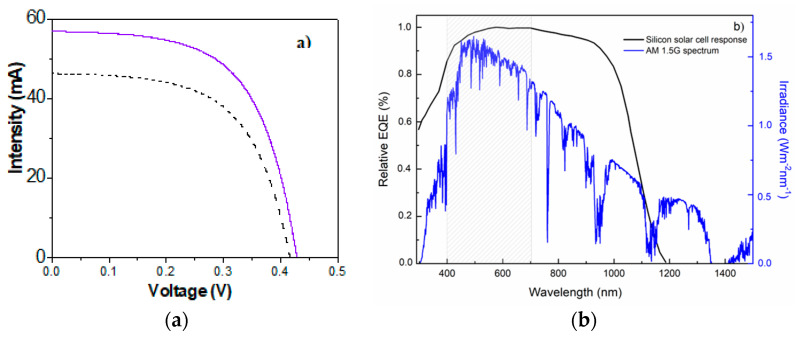
(**a**) Current voltage curves for the composite AND/AlqCl film (solid violet line) and the AND support (black dashed line). (**b**) Wavelength dependence of external quantum efficiency (black line, left side y-exe) and irradiance (blue line, right side y-exe).

Consequently, the I-V curves of the silicon solar cell covered by the composite AND/AlqCl film and the AND support were measured, and the results are indicated in Figure 8a. These results show the increase in the current for the AND/AlqCl film when it is compared with the nanoporous alumina AND support. The explanation for this behaviour requires a combined reading of transmission/reflection measurements (Figure 6) and the external quantum efficiency (EQE) of the used solar cell (Figure 8b). As seen in the transmission spectrum (Figure 6a), there is a remarkable increment in the value of light transmission in the visible range (400 nm to 800 nm) for the AND/AlqCl film compared to the AND support. In addition, a noticeable reduction in the reflectance values of the composite AND/AlqCl film for that wavelength interval of frequency was also obtained (Figure 6b). Therefore, when the AND/AlqCl sample is placed over the solar cell, more solar radiation reaches the solar cell than when only the AND support is present. Furthermore, this increase occurs in the wavelength range with more light power, and just in the response range of the solar cell, as shown in Figure 8b. Consequently, values of the short-circuit current (I_sc_) and the power at the maximum power point (P_mp_) of the I-V curve will increase as more solar radiation reaches the solar cell, allowing it to generate more photocurrent. Table 2 shows the values of I_sc_ and P_mp_, as well as those for typical solar cell parameters: open circuit voltage (V_oc_), fill factor (FF), and efficiency (E_ff_) obtained for the composite AND/AlqCl and the AND films. These values are practically the same as those obtained for the composite film obtained covering the AND support with the IL OMIMPF_6_ (I_sc_ = 57.27 mA and P_mp_ = 14.74 mW), but slightly higher than for other imidazolium-based ILs [30].

## 4. Conclusions

Surface coverage by the ionic liquid AliquatCl of an asymmetric nanoporous alumina structure (AND) clearly modified different optical characteristic parameters (refraction index, extinction coefficient, dielectric constant, and photoluminescence). The average value of the refraction index determined for the composite AND/AlqCl thin film by spectroscopic ellipsometric measurements (wavelength between 250 and 1250 nm) confirms the complete coverage of the support, in agreement with the results obtained by the XPS measurements, which also indicate AlqCl percolation through the AND nanopores. Moreover, angle-resolved XPS analysis shows the uniformity of the thin top layer of the composite sample. 

Both the significant increase in the light transmission percentage and the reduction in light reflection in the visible region exhibited by the composite AND/AlqCl thin film with respect to the nanoporous AND support, which depend on the liquids covering the support surface, are two points of great interest due to the accessibility and wide range of applications of these optical measurements. The effect on the photoluminescence spectrum of the nanoporous alumina support associated with its coverage with the IL AlqCl IL, an efficient increase of 29% when placed over the silicon solar cell, is another relevant point. This behaviour is a clear indication of its potential when used in renewable energy applications, particularly in solar cell technologies.

## Figures and Tables

**Table 1:** Atomic concentration percentages and concentration ratios of the different elements found on both surfaces of the AND/AlqCl and AND thin films and the IL AlqCl (C_25_H_54_ClN).

Sample	C (%)	N (%)	Cl (%)	Al (%)	O (%)	C/Cl	N/Cl	O/Al
AND/AlqCl ^a^	79.8	3.1	2.8	4.4	7.7	28.5	1.11	1.75
AND/AlqCl(op)	57.7	1.9	1.6	15.3	23.5	38.5	1.33	1.53
AND ^b^	16.5	0.4	—	27.5	51.9	—	—	1.85
AND(op) ^c^	17.3	0.6	—	28.4	50.4	—	—	1.77
AlqCl	93.2	3.5	3.3	—	—	28.2	1.06	—

^a^ Si: 1.9%; ^b^ P: 3.1%; ^c^ P: 3.3%.

**Table 2:** Average values of the short-circuit current (I_sc_), power at the maximum power point (P_mp_), open circuit voltage (V_oc_), fill factor (FF), and efficiency (E_ff_) percentages.

Sample	I_sc_ (mA)	P_mp_ (mW)	V_oc_ (mV)	FF (%)	E_ff_ (%)
NPAS	46.41	11.41	0.417	59.9	6.46
AND/AlqCl	57.02	14.71	0.429	60.2	8.33

## Data Availability

The original contributions presented in the study are included in the article/Supplementary Material, further inquiries can be directed to the corresponding author.

## Supplementary Materials

The following supporting information can be downloaded at: https://www.mdpi.com/article/10.3390/mi15060739/s1, Figure S1: SEM micrographs of AND alumina support surfaces; Figure S2: Scheme of spectroscopy ellipsometry measurements; Figure S3: Light transmission and reflection as a function of wavelength for the AND support and the AND support covered with the IL AlqCl, the IL OMIMPF_6_, and distilled water.
