# Supplementary material for: Optical Modification of a Nanoporous Alumina Structure Associated with Surface Coverage by the Ionic Liquid AliquatCl
Source: Micromachines (Basel). 2024 May 31;15(6):739. doi: 10.3390/mi15060739 (PMC11206012; doi:10.3390/mi15060739)
Supplement: Supplementary file 1 [file micromachines-15-00739-s001.zip › micromachines-2974023-supplementary.pdf]

Supplementary Information: Optical Modification of a Nanoporous Alumina Structure Associated with Surface Coverage by the Ionic Liquid AliquatCl

María Cruz López-Escalante [1], Valle Martínez de Yuso [2], Ana L. Cuevas [3] and Juana Benavente [4,*]

1 The Nanotech Unit, Laboratorio de Materiales y Superficies, Departamento de Ingeniería Química, Facultad de Ciencias, Universidad de Málaga, 29071 Málaga, Spain; mclopez@uma.es
2 Lab. de Espectroscopía de Rayos X, Servicios Centrales de Apoyo a la Investigación (SCAI), Universidad de Málaga, 29071 Málaga, Spain; mvyuso@uma.es
3 Unidad de Nanotecnología, Servicios Centrales de Apoyo a la Investigación (SCAI), Universidad de Málaga, 29071 Málaga, Spain; analaura.cuevas@uma.es
4 Departamento de Física Aplicada I, Facultad de Ciencias, Universidad de Málaga, 29071 Málaga, Spain
* Correspondence: j_benavente@uma.es

(a) (b)

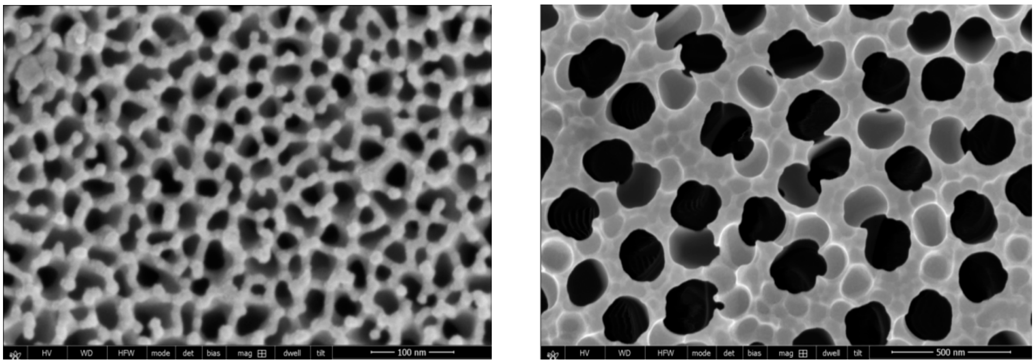

Figure S1: SEM micrographs of: (a) top (denser) and (b) bottom (opener) surfaces of the AND alumina support.

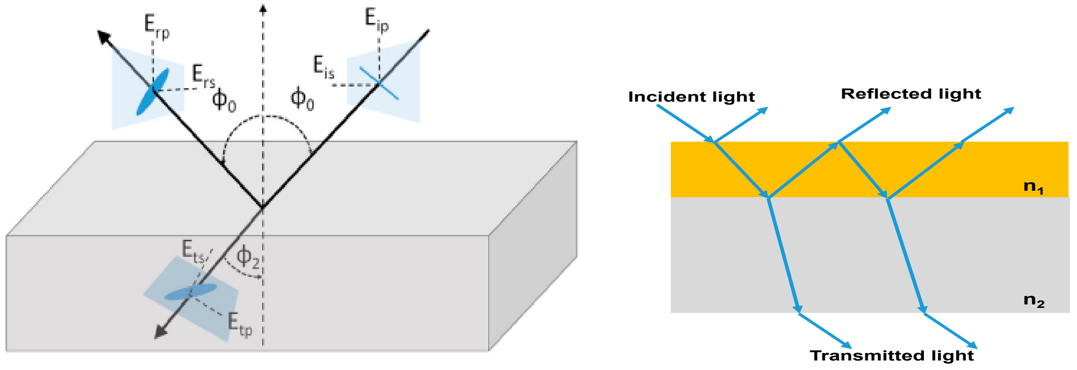

(a) (b)

Figure S2. Scheme of spectroscopy ellipsometry measurement for: **(a)** homogeneous sample; **(b)** surface monolayer coated sample

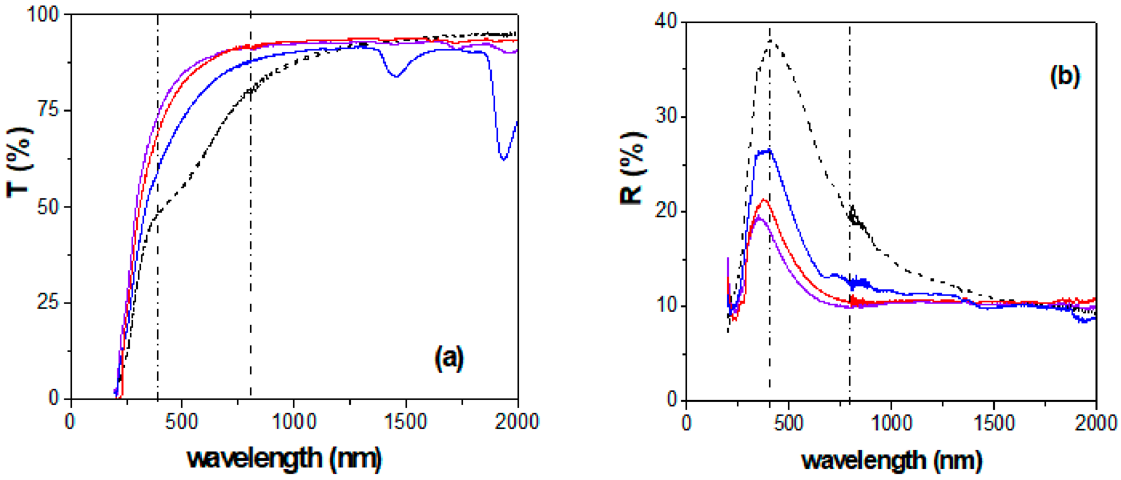

Figure S3: Light transmission (a) and reflection (b) percentages as a function of wavelength. AND support (black dashed line); composite AND/AlqCl film (solid violent line); AND support covered with the IL OMIMPF6 ($C_{12}H_{23}N_2.PF_6$) (red solid line) and AND support covered with distilled water (blue solid line).
